# Aging, Fitness, and Marathon Times in a 91 Year-old Man Who Competed in 627 Marathons

**DOI:** 10.9734/BJMMR/2015/17946

**Published:** 2015-06-08

**Authors:** Odessa Addison, Gregory Steinbrenner, Andrew P. Goldberg, Leslie I. Katzel

**Affiliations:** 1 Division of Gerontology and Geriatric Medicine, University of Maryland School of Medicine, USA.; 2 The Baltimore Veteran Affairs Medical Center, Geriatrics Research Education and Clinical Center, Baltimore, Maryland 21201, USA.

**Keywords:** Maximal aerobic capacity, longitudinal, exercise, athlete

## Abstract

Aging is associated with a decline in maximal aerobic capacity (VO_2max_) that may be attenuated by chronic endurance exercise. This case study chronicles the changes in marathon times in a 91 year old man who completed 627 marathons and 117 ultramarathons over 42 years. He began running marathons at age 48. His yearly best times remained fairly constant at ~240 minutes from age 50 – 64 years and then gradually rose to about 260 minutes in his early seventies followed by a curvilinear deterioration as he approached his ninth decade. His times plateaued at ~ 600 minutes in his late eighties. Between ages 68 and 89 his VO_2max_ declined from 43 to 20 ml/kg/min. His marathon times were highly correlated with his VO_2max_ (r^2^=0.87). The decline in marathons times and VO_2max_ may reflect the contributions of biological aging, changes in exercise training volume and intensity, injuries, and comorbid disease.

## 1. INTRODUCTION

Maximal oxygen uptake or VO_2max_ declines with age [1–3] and is associated with reductions in functional capacity [4], as well as increased mortality risk [5]. In older adults the rate of decline in VO_2max_ accelerates with age, representing a curvilinear decline and greater absolute reductions in fitness [6–9]. Factors underlying this age-associated reduction in VO_2max_ may include decreased muscle mass [2], declines in skeletal muscle mitochondrial function [10], reduced cardiac output [11], and decreased physical activity [7]. Many hypothesize that the age-associated decline in VO_2max_ is attenuated in endurance trained athletes who continue habitual exercise [3–5,12,13]. However, the data is equivocal as some studies find no significant difference in absolute VO_2max_ declines with age in athletes compared to sedentary peers [14], while others find greater absolute declines compared to sedentary individuals [2,3,15]. This case report documents the changes in VO_2max_ and health in a master athlete over 20 years.

## 2. PRESENTATION OF CASE

We previously reported the changes in marathon performance and VO_2max_ in this male, who at age 81 had competed in 591 marathons and ultramarathons [16]. We continued to follow this individual for an additional decade, and to provide greater clinical insight into the longitudinal changes in VO_2max_ with aging, in this report we chronicle 4 decades of marathon times and the changes in VO_2max_ over 23 years.

### 2.1 Participant

The University of Maryland Institutional Review Board approved this study, and the subject provided informed consent. The patient, was initially seen at the age of 68 and was subsequently studied at our facility 12 times over 23 years to measure his VO_2max_. He agreed to the use of his data for this case report. Maximal exercise treadmill tests with measurement of VO_2max_ were performed at each time point. The VO_2max_ was determined using a protocol as previously described [16].

### 2.2 Training History and Marathon times

The patient kept logs of his weekly training mileage until age 81. Based on his self-report records, he completed 627 marathons and 117 ultramarathons. We analyzed times from 623 marathons in this manuscript, but did not analyze the times from the 117 ultramarathons as these covered a variety of distances and times (i.e. 50 kilometers, 50 miles, 100 kilometers, 24 hours, etc.) and required different running paces. We do however, report the number of ultramarathons completed each year. Pearson product moment correlation coefficients were calculated for his VO2 tests and marathon times using SPSS 22 (Armonk, New York).

## 3. RESULTS

**Fig. 1:**
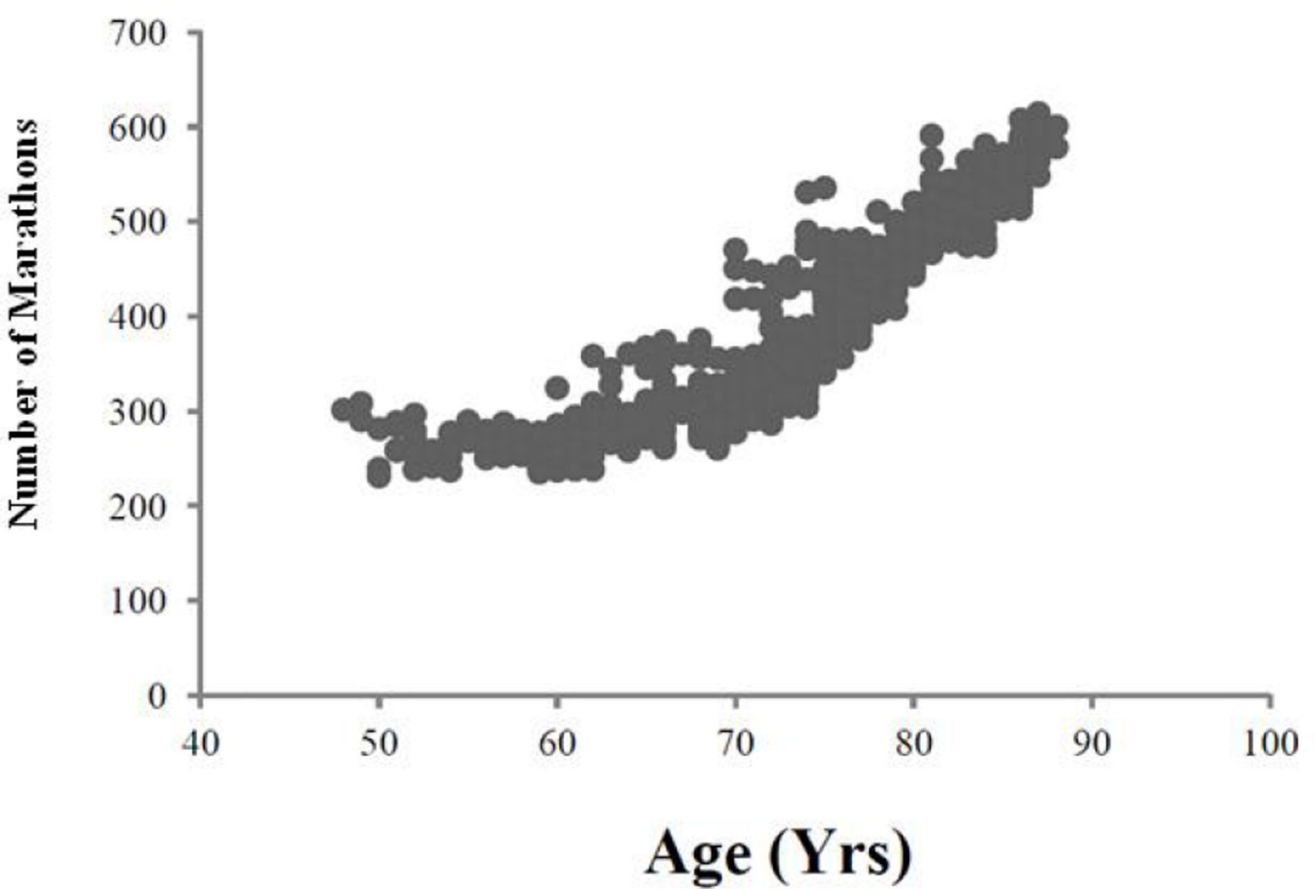
There is a curvaliner increase in this patient’s marathon times as he approached his ninth decade of life

The patient ran his first marathon, the Boston Marathon, at age 48 in 1969. During his first decade of training there was a decrease in his marathon times and he achieved his personal lifetime best marathon time of 231 minutes at age 51 years (Fig. 1). His yearly best times remained fairly constant at ~240 minutes until 64 years and then gradually rose to ~260 minutes in his early seventies. As he approached his eighties, there was a curvilinear deterioration in his marathon times with a plateau at ~600 minutes in his late eighties.

**Fig. 2:**
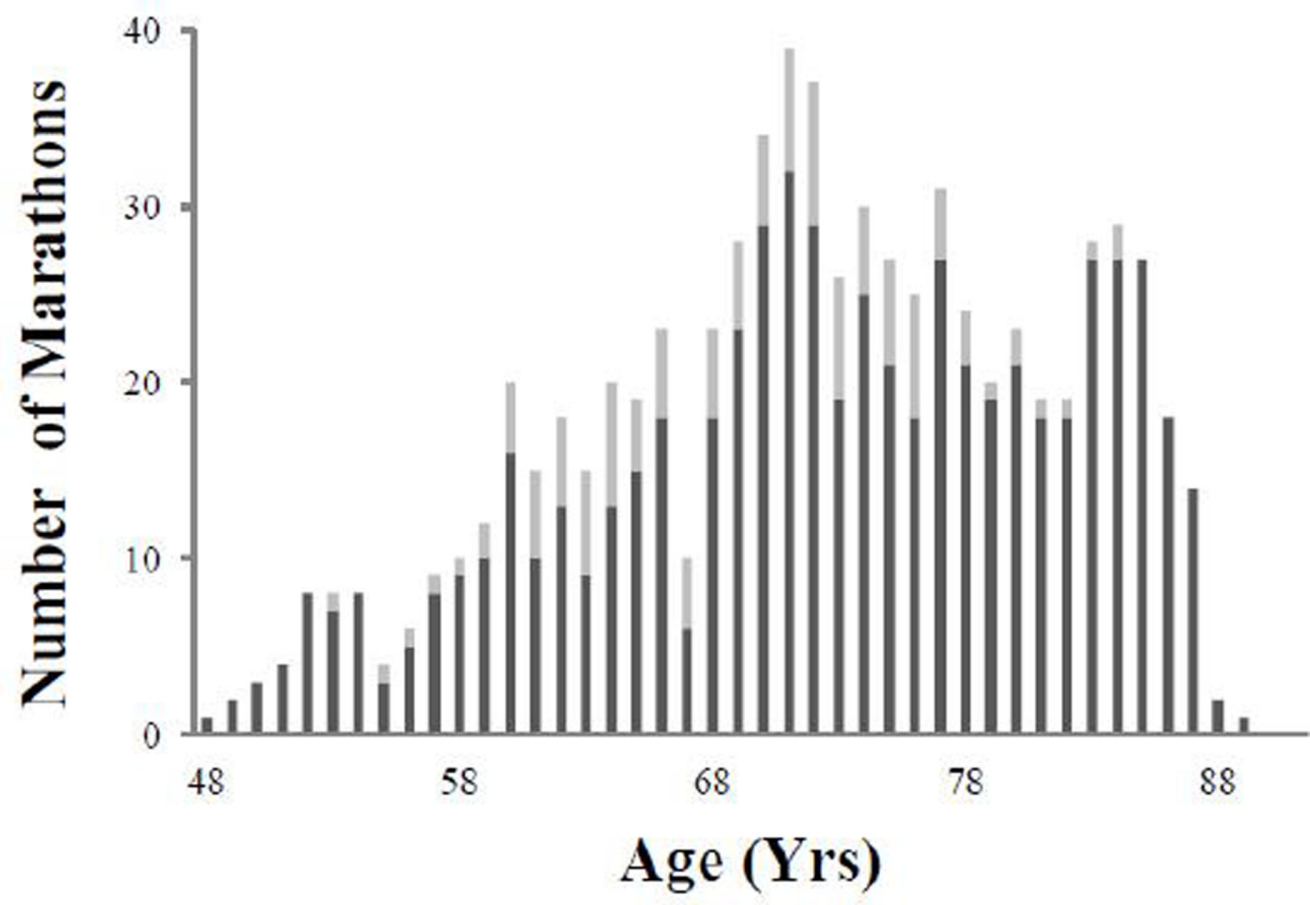
Number of marathons and ultramarathons competed in per year. He was able to compete on average in >20 marathons a year between ages 69 and 87 years with a drop off at age 88. Marathons in dark bar, ultramarthons in line bar

His lifetime goal was not to run the fastest marathons but rather to complete the greatest number of marathons, averaging ~2/month in 18 countries and 6 continents. The number of marathons that he competed in increased from ~ 6/year in his first 10-years of running to about 20/year in his third-decade of running (Fig. 2). At age 67 he had a radical prostatectomy which accounted for the low number of marathons run that year. The number of marathons he competed in in one year peaked at ~30/year in his early seventies. From 70–80 years he competed in 295 marathons and 58 ultramathons, and in his eighties he competed in 177 marathons and 7 ultramarathons. His last ultramarthon was at age 84. Average weekly training mileages for the first three decades of competition were 28–33 miles/week and his training volume negatively correlated with race times across each decade. (r = −0.64, P<0.001; r = −0.66, P<0.001; r = −0.88, P<0.001).

**Fig. 3:**
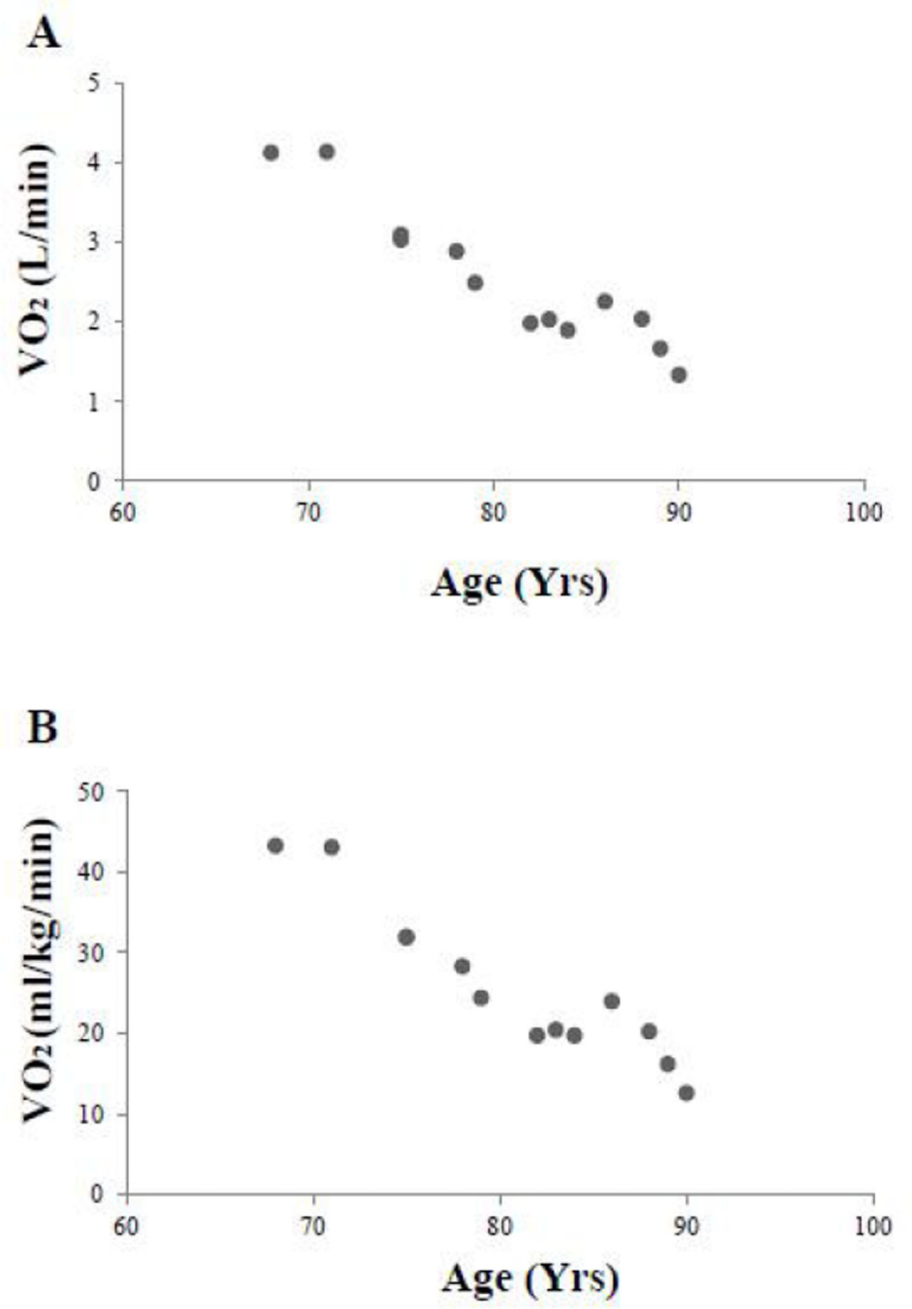
VO_2max_ versus age: (A) in L/min; (B) in ml/kg/min. He maintained his VO_2max_ during the 4 years between the first two tests with a subsequent decline as he transitioned from running to walking. Similar results are noted when VO_2max_ was expressed in either L/min (panel A) or adjusted for total body weight (panel B)

**Fig. 4:**
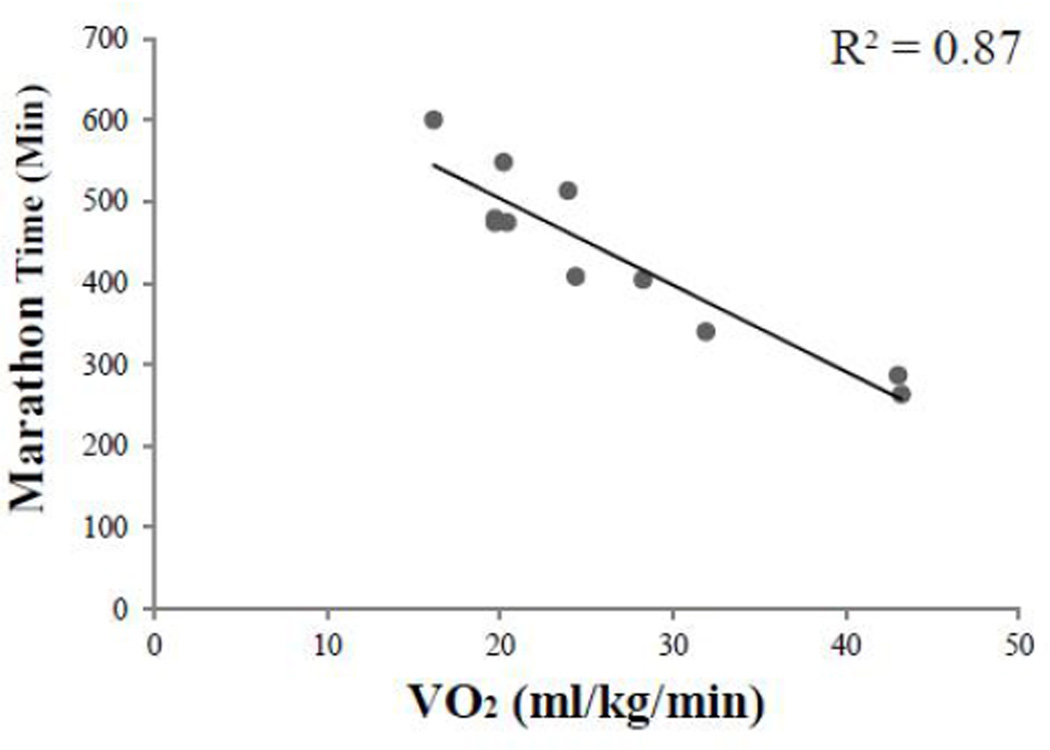
Relationship of VO_2max_ to best yearly marathon time for that age. His marathon times over the 23 years were strongly related to VO_2max_ (r2=0.87, p < 0.01)

His VO_2max_ at ages 68–72 years was approximately 43 ml/kg/min with an average marathon time ~260 minutes that then declined over the next 23 years (Fig. 3). His marathon times over the next 23 years were highly correlated with VO_2max_ (r2=0.87, p<0.01) (Fig. 4). In his mid-seventies he suffered multiple injuries including: plantar fasciitis, lower back pain, and fracture of multiple ribs as a result of a fall that led to a greater focus on walking. His VO_2max_ continued to decline in his eighties when he developed asymptomatic atrial fibrillation that was medically converted and maintained pharmacologically in normal sinus rhythm. When he was in his late eighties he was diagnosed with asymptomatic chronic lymphocytic leukemia, but did not require pharmacological therapy. Remarkably even with these well controlled but chronic medical problems he was able to complete 14-marathons at age 87. He further completed two marathons at age 88, and 1 at age 89. Shortly after turning 90 he entered his final marathon, but dropped out after 12 miles. After this he stopped competing in marathons, saying that he had lost his motivation. He was satisfied with his lifetime accomplishment completing 627 marathons and 117 ultramarathons. Overall his VO_2max_ declined by 70% over this 23 year longitudinal study.

## 4. DISCUSSION

This case report offers a unique perspective on successful aging. We chronicle the longitudinal age-associated declines in marathon times and VO_2max_ in a 91-year old man who competed in 627 marathons and 117 ultra marathons over a 42 year period of time.

Initially his times improved between ages 48 and 51, followed by modest increases in his times over the 22 year period between 51 – 73 years. The subsequent deterioration in his times appeared to follow a curvilinear pattern with a plateau and further gradual deterioration as he approached 90 years of age. His marked declines in VO_2max_ were associated with dramatic declines in performance in older age. Remarkably even as his VO_2max_ (ml/kg/min) declined to the low 20s he was still able to walk for > 9 hours to complete the marathons.

His main goal was to participate in the greatest number of marathons rather than achieving the best possible times. This often necessitated participation in several marathons on consecutive days (3-in-10 days), rather than spacing them out over a longer time period. As best we can determine, this patient competed in the most marathons for anyone age > 70 years. While habitual physical activity results in improved metabolic and general health [17], marathon running may also result in transient increases in cardiac biomarkers of injury and systolic dysfunction [18]. Given the physical toll that marathons place on the body, it is likely that if he competed in fewer races per year he may have substantially shortened his race times.

The longitudinal age-associated declines in VO_2max_ and marathon times in this master athlete were not linear and may reflect a combination of the contributions of aging, decreases in training, injuries and comorbid conditions that occurred over 20 years of this report. Hoffman and Parise [19] recently reported that high level performances in ultra-marathon runners can be sustained late into the 4^th^ decade, however further aging is associated with declines in performance times. Between ages 72 and 80 our patient’s VO_2max_ declined by 44%, from 43 to 24 ml/kg/min. Between 80 and 90 years his VO_2max_ declined an additional 16% (4 ml/kg/min). A major factor that appears to have contributed to his longitudinal decline in VO2max was his inability to continue running as he approached 80 years of age.

Previous longitudinal studies show that master athletes experience declines in VO_2max_ as their training regimens decreased, but the magnitude of the decline is variable [13,15,20]. Robinson et al. reported a mean VO_2max_ decline from 71.4 ml/kg/min to 41.8 ml/kg/min in 13 former champion runners [20]. Trappe et al. [13] studied a cohort of endurance runners with a mean age of 68 years old who reduced their workout to non-competitive physical activity, and yet there was only a 33% decline over 22 years or ~15% per decade. In contrast, Pollock et al. [21] showed a larger (~34%) decline in VO_2max_ over 10 years in a low intensity trained group of track athletes. We previously reported longitudinal data on the change in 42 athletes from an original cohort of 70 athletes [22]. We followed and studied 18 athletes 3 times over an average of 14 years. There were substantial longitudinal reductions in VO_2max_ as they reduced their training levels over 8 years and cumulatively there was an overall 37% decline in VO_2max_ over 14 years. Based on their self-reported training records, only 10 of the 18 athletes were still running (averaging 20 miles per week) and none were training at comparable running paces with their initial evaluation. This 37% decline is far greater than the 15% loss in VO_2max_ that would have been expected if cross-sectional data from endurance trained athletes were used to predict losses over a similar 14 year period [8]. It has been suggested that athletes experience greater rates of decline in maximal aerobic capacity then sedentary peers [23]. One suggested explanation is an inability to maintain a high training intensity may result in an accelerated drop off in performance and VO_2max_ even in successful aging older ex-athletes [9,23].

Buskirk and Hodgson [1] introduced the concept of a curvilinear relationship between VO_2max_ and age. The curvilinear relationship between VO_2max_ and age may explain the curvilinear relationship between age and running performance [12]. In this patient there was a direct relationship between VO_2max_ and marathon times, which accounted for 87% of the variance in his marathon times. Presumably the increased rate of decline in fitness in the 8th and 9th decades of life may be related in part to his reduction in training intensity and volume [21,24], but a number of other factors may contribute to the accelerated decline including primary biologic aging. Reductions in stroke volume and maximal heart rate strongly impact VO_2max_, and may increase the rate of decline in VO_2max_ with aging [9,24]. Other factors that may influence declines in VO_2max_ include declines in arterial-venous oxygen uptake due to reductions in skeletal muscle capillary density and mitochondrial O2 and the efficiency of central and peripheral oxygen uptake [12–14,21]. The strengths of this observational case report include the extensive longitudinal data detailing hundreds of marathon times spanning four decades that are accompanied by objective VO_2max_ measurements performed over a 23-year period of time. The additional 10 years of data presented in the case report suggest that the decline in functional performance and VO_2max_ may reflect the physiological contributions of biologic aging, decreased training volume and intensity, injuries and comorbid disease. One limitation of this case report is our reliance on the patients self-reported race times and training volume. However, we were able to spot check many of the times he provided and found that the reported race times were accurate. Although we have training records till age 81, we lack detailed training records as he approached the end of his career at age 90. Nevertheless over the first 23-years of follow-up we show significant relationships between miles trained per week and marathon times, hence it is likely that similar results would be obtained if we extended this for an additional decade. While VO_2max_ measurements are highly informative, we also lack more mechanistic measurements of central cardiac output, peripheral blood flow, and mitochondrial function would provide additional insight into the mechanisms that contribute to declines in fitness and performance. Finally, there are limits to the degree in which our observations from this one individual can be generalized to changes in fitness and performance in oldest age. Certainly the curvilinear pattern that we and others have observed [6,7] suggests there are inherent effects and limitations imposed by biologic aging on cardiac and muscle function.

## 5. CONCLUSION

In summary, this 91-year old man competed in 627 marathons and 117 ultramarathons over a 42 year period of time. The longitudinal decline in functional performance and VO_2max_ in this master athlete may reflect the contributions of biologic aging, decreased training volume and intensity, injuries and comorbid medical disease.
